# Value of Sonazoid-enhanced ultrasonography in characterizing indeterminate focal liver lesions on gadoxetic acid-enhanced liver MRI in patients without risk factors for hepatocellular carcinoma

**DOI:** 10.1371/journal.pone.0304352

**Published:** 2024-05-24

**Authors:** Ji Yoon Ban, Tae Wook Kang, Woo Kyoung Jeong, Min Woo Lee, Boram Park, Kyoung Doo Song

**Affiliations:** 1 Department of Radiology, Samsung Medical Center, Sungkyunkwan University, Seoul, Korea; 2 Biomedical Statistics Center, Research Institute for Future Medicine, Samsung Medical Center, Seoul, Korea; Yonsei University College of Medicine, REPUBLIC OF KOREA

## Abstract

**Purpose:**

To evaluate the added value of contrast-enhanced ultrasonography (CEUS) using Sonazoid in characterizing focal liver lesions (FLLs) with indeterminate findings on gadoxetic acid-enhanced liver MRI in patients without risk factors for hepatocellular carcinoma (HCC).

**Methods:**

Patients who underwent CEUS using Sonazoid for characterizing indeterminate FLLs on gadoxetic acid-enhanced liver MRI were. The indeterminate FLLs were classified according to the degree of malignancy on a 5-point scale on MRI and combined MRI and CEUS. The final diagnosis was made either pathologically or based on more than one-year follow-up. The diagnostic performance was assessed using a receiver operating characteristic (ROC) curve analysis, and the net reclassification improvement (NRI) was calculated.

**Results:**

A total of 97 patients (mean age, 49 years ± 16, 41 men, 80 benign and 17 malignant lesions) were included. When CEUS was added to MRI, the area under the ROC curve increased, but the difference was not statistically significant (0.87 [95% confidence interval {CI}, 0.77–0.98] for MRI vs 0.93 [95% CI, 0.87–0.99] for CEUS added to MRI, *P* = 0.296). The overall NRI was 0.473 (95% CI, 0.100–0.845; *P* = 0.013): 33.8% (27/80) of benign lesions and 41.2% (7/17) of malignant lesions were appropriately reclassified, whereas 10.0% (8/80) of benign lesions and 17.6% (3/17) of malignant lesions were incorrectly reclassified.

**Conclusions:**

Although performing CEUS with Sonazoid did not significantly improve the overall diagnostic performance in characterizing indeterminate FLLs on gadoxetic acid-enhanced liver MRI in patients without risk factors for HCC, it may increase radiologist’s confidence in classifying FLLs.

## Introduction

Contrast-enhanced ultrasonography (CEUS) is an imaging modality that uses ultrasound contrast agents (USCAs), which are excreted by the lungs; thus, they can be administered to patients with impaired kidney function without the risk of contrast-induced nephropathy [1]. Sonazoid (GE Healthcare, Oslo, Norway), a second-generation USCA, consists of lipid-coated perfluorobutane gas microspheres [2] and is phagocytosed by Kupffer cells (liver-specific macrophages); thus, performing CEUS using Sonazoid enables the imaging of the Kupffer phase in addition to the vascular-phase [3]. In the Kupffer phase, obtained 10 to 15 minutes after contrast injection, the absence of normal Kupffer cells appears as a defect on the image [4].

Performing CEUS using Sonazoid can provide Kupffer phase images and enable real-time image acquisition, providing additional information that cannot be obtained using CT or MRI. Indeed, several studies have shown the additional value of CEUS using Sonazoid for diagnosing hepatocellular carcinoma (HCC) [5, 6]. Therefore, the Asian Pacific Association for the Study of the Liver guidelines recommend the use of CEUS with Sonazoid to diagnose HCC in observations with atypical enhancement patterns on CT or MRI [7].

However, most studies have investigated the usefulness of performing CEUS using Sonazoid in the evaluation of focal liver lesions (FLLs) in patients with risk factors for HCC. The World Federation for Ultrasound in Medicine and Biology guidelines recommend CEUS for characterizing FLLs in non-cirrhotic livers that are indeterminate on CT or MRI [8]. However, there is limited evidence supporting this recommendation. Therefore, we aim to evaluate the added value of CEUS using Sonazoid in characterizing FLLs with indeterminate imaging findings on gadoxetic acid-enhanced liver MRI in patients without risk factor for HCC.

## Materials and methods

This retrospective study was approved by the Institutional Review Board of Samsung Medical Center). The requirement for an informed consent was waived because the study involved a retrospective review of medical records and images. The data were accessed from January 1, 2023 to June 31, 2023 for research purposes. The first author and corresponding author had access to information that could identify individual participants during data collection.

### Patients

**Fig 1:**
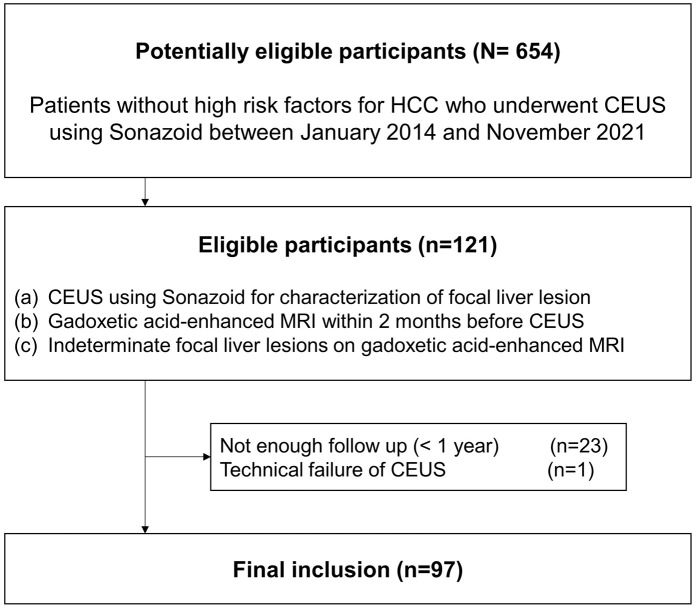
Inclusion flow chart.

Between January 2014 and December 2021, patients who fulfilled the following eligibility criteria were consecutively enrolled in our study: (1) underwent CEUS using Sonazoid for characterizing of FLLs, (2) underwent gadoxetic acid-enhanced liver MRI within 2 months before undergoing CEUS, (3) showed indeterminate FLLs on gadoxetic acid-enhanced liver MRI, (4) had no high-risk factors (cirrhosis caused by chronic viral hepatitis B and C, chronic alcohol abuse) for HCC. Indeterminate FLLs were defined as lesions difficult to characterize accurately using MRI as they did not show the typical imaging findings of benign or malignant hepatic lesions. A radiologic resident (blinded) reviewed standardized radiological reports and gadoxetic acid-enhanced liver MRI images and selected patients who showed indeterminate FLLs on gadoxetic acid-enhanced liver MRI. Subsequently, a radiologist (blinded, with 14 years of experience in gastrointestinal imaging) reviewed the gadoxetic acid-enhanced liver MRIs of the selected patients and confirmed the presence of indeterminate FLLs. Patients with inadequate-quality CEUS images and those lost to follow-up within 1 year after CEUS were excluded. Fig 1 summarizes the patient-selection process.

### Image acquisition

MRI scans were acquired using a 3.0-T whole-body MR system (Intera Achieva 3.0-T [Philips Healthcare, Best, The Netherlands] and Magnetom Skyra [Siemens Healthcare, Erlangen, Germany]). The liver MRI protocol was composed of T1-weighted in- and out-of-phase images, breath-hold multi-shot T2-weighted images, respiratory-triggered single-shot T2-weighted images, respiratory-triggered single-shot heavily T2-weighted images, dynamic enhanced T1-weighted images, and diffusion-weighted images with b-values of 0, 100, and 800 s/mm^2^. Regarding contrast-enhanced imaging, unenhanced, arterial-phase (20–35 s), portal-phase (60 s), transitional-phase (3 min), and hepatobiliary-phase (20 min) images were acquired. Regarding arterial-phase imaging, the timing of image acquisition was determined using the MRI fluoroscopic bolus detection technique. An intravenous contrast agent (Primovist; Bayer Schering Pharma, Berlin, Germany) was injected at a rate of 2 mL/s with a total dose of 0.025 mmol/kg body weight, followed by a 20-mL saline flush.

All CEUS examinations were performed by abdominal radiologists with at least 10 years of clinical experience in CEUS. CEUS was performed with a 1–5 or 1–7 MHz convex probe using LOGIQ E9 (GE Healthcare, Milwaukee, WI, USA) or RS80A (Samsung Medison, Seoul, Korea) ultrasound systems. Ultrasound-MRI fusion was routinely performed by using Volume Navigation (GE Healthcare) or S-fusion (Samsung Medison) systems to accurately localize lesions. Then, CEUS was performed with either B mode and contrast mode US, or contrast mode US and fused MR images on a dual-screen display. Contrast harmonic imaging was performed using a default mechanical index setting of 0.20–0.26. The dynamic range was 60–65dB. The beam was focused at the posterior margin of the liver. Sonazoid was intravenously administered into the antecubital vein at a dose of 0.015 mL/kg body weight, followed by a 10 mL saline flush. Arterial-, portal venous-, late vascular-, and postvascular-phase (i.e., Kupffer phase) images were obtained approximately at 10–40 seconds, 60–90 seconds, 3–4 minutes, and 10 minutes, respectively after the contrast agent injection.

### Image evaluation

At the first session, one radiologist (blinded, radiologist with 14 years of experience in gastrointestinal imaging and 10 years of experience in CEUS), who did not perform the CEUS included in this study, reviewed the MRIs first blind to the results of CEUS and the final diagnosis of the FLLs. A CEUS operator group (blinded, radiologists with more than 15 years of experience in gastrointestinal imaging and 11 years of experience in CEUS at the time of image review) who performed the CEUSs included in this study reviewed the MRIs of patients for whom they performed the CEUSs independently. The target indeterminate FLLs were categorized into following five categories based on the degree of benignity or malignancy: probably benign, more likely benign, indeterminate, more likely malignant, and probably malignant. All the lesions included in this study were indeterminate lesions that did not show typical imaging findings of specific benign or malignant lesions on MRI. The categorization of these lesions was based on a comprehensive assessment of the overall MR imaging findings [9, 10].

At the second session, conducted at an interval of one month, one radiologist (blinded) and a CEUS operator group reviewed the CEUS with MRIs; however, they were blinded to the MRI categories assessed at the first session. Again, the target indeterminate FLLs were categorized into five categories based on the degree of benignity or malignancy. The CEUS findings used for the categorization of FLLs were the imaging features of each individual lesion previously reported [8, 11]. If a lesion showed characteristic imaging features of a specific benign or malignant lesion, it was classified accordingly. If it did not show typical features, the presence of the following imaging characteristics was considered indicative of a higher possibility of malignancy: arterial phase hyper-enhancement (rim-like enhancement, complete enhancement, inhomogeneous enhancement) and any degree of washout on portal venous, late vascular phase, or Kupffer phase.

After the two session, the final categories of FLLs on MRI and CEUS added to MRI were determined by consensus between the radiologist (blinded) and the CEUS operator group. When there was agreement between the categories stated by the radiologist and those stated by the CEUS operator group, the corresponding category was used. For cases with disagreement, the two groups reviewed the images again and exchanged their opinions to reach a consensus.

### Reference standard

Electronic medical records and follow-up images were reviewed to determine the reference standards. An FLL was considered malignant if the lesion was pathologically diagnosed as malignant. An FLL was considered benign if the lesion was pathologically diagnosed as benign, the lesion disappeared during follow-up, or no changes occurred in 1 year.

### Statistical analysis

An inter-observer agreement between the radiologist (blinded) and the CEUS operator group for the categories of indeterminate FLLs on MRI and CEUS added to MRI was evaluated using the weighted Kappa statistics. The Kappa value interpretations were as follows: κ ≤ 0.20, slight; 0.2 < κ ≤ 4, fair; 0.4 < κ ≤ 0.6, moderate; 0.6 < κ ≤ 0.8, good; and 0.8 < κ ≤ 1.0, excellent. To evaluate the diagnostic performance, a receiver operating characteristic (ROC) curve was plotted, and the area under a ROC curve (AUC) was calculated. The DeLong’s test was used to test whether the AUCs of the two models were significantly different. The net reclassification improvements (NRI) was calculated to assess the improvement of diagnostic classification by adding the CEUS. In malignant FLLs, the NRI was calculated as (proportion of FLLs that were reclassified into a higher category of malignancy–proportion of FLLs that were reclassified into a lower category of malignancy). In benign FLLs, the NRI was calculated as (proportion of FLLs that were reclassified into a lower category of malignancy–proportion of FLLs that were reclassified into a higher category of malignancy). The overall NRI was calculated as the NRI of malignant FLLs + the NRI of benign FLLs. The Sankey diagram was presented to visualize the flow between the two diagnostic methods. Results were considered statistically significant when the two-sided p-values were less than 0.05. All analyses were performed using the SAS software (version 9.4, SAS Institute Inc., Cary, NC, USA.), and the R software (version 4.0.5, R Project for Statistical Computing).

## Results

A total of 97 patients (80 with benign FLL and 17 with malignant FLL) were included. The baseline characteristics of the patients and their hepatic lesions are summarized in Table 1. Malignant FLLs included HCC (n = 12), cholangiocarcinoma (n = 1), epithelioid hemangioendothelioma (n = 1), lymphoma (n = 1), metastatic meningioma (n = 1), and metastatic colon cancer (n = 1). Of the 80 benign FLLs, 29 were pathologically confirmed: adenoma (n = 9), focal nodular hyperplasia (n = 4), hemangioma (n = 4), angiomyolipoma (n = 3), eosinophilic abscess (n = 3), necrotic nodule (n = 2), reactive lymphoid hyperplasia (n = 2), biliary hamartoma (n = 1), and schwannoma (n = 1).

**Table 1:** Characteristics of included patients.

Characteristics	Included Patients (N = 97)
Age (years)	49 ± 16
Sex (male)	41 (42.3%)
Underlying malignancy (yes)	19 (19.6%)
Size of FLL (cm)	2.3 ± 1.5
Final diagnosis of FLL (malignancy)	17 (17.5%)
MR finding of FLL	
APHE	85 (87.6%)
Low SI on HBP	65 (67.0%)
Restricted diffusion	29 (29.9%)
Mild-moderate high SI on T2WI	74 (76.3%)
Washout on portal venous phase	28 (28.9%)
Fat in FLL	9 (9.3%)

Note.―The data are mean ± standard deviations and the number of patients with percentage in parentheses. FLL = focal liver lesion, APHE = arterial phase hyperenhancement, SI = signal intensity, HBP = hepatobiliary phase, T2WI = T2 weighted image.

Among 17 malignant FLLs, 16 (94%) lesions showed hypo-enhancement on the Kupffer phase, and one (6%) lesion showed iso-enhancement. Among 80 benign FLLs, 30 (38%) lesions showed hypo-enhancement, 40 (50%) lesions showed iso-enhancement, and 10 (13%) lesions showed hyper-enhancement.

### Interobserver agreement for the FLL categories

The interobserver agreement between the two groups for categories of the FLLs was good (κ = 0.783, 95% confidence interval [CI]: 0.693–0.874) on MRI and excellent (κ = 0.909, 95% CI: 0.858–0.959) on CEUS added to MRI.

### Diagnostic performance

When CEUS was added to MRI, the AUC for characterizing indeterminate FLLs increased, but the difference was not statistically significant (0.87, 95% CI: 0.77–0.98 for MRI vs 0.93, 95% CI: 0.87–0.99 for CEUS added to MRI, *P* = 0.296) (Table 2).

**Table 2:** Results of AUC analysis.

	N	Malignancy (%)	Model	AUC			
				AUC value	95% CI	Difference	*P* value*
**Total**	97	17 (17.5)					
			MRI	0.87	[0.77, 0.98]		
			CEUS + MRI	0.93	[0.87, 0.99]	0.05	0.296
**Underlying extrahepatic malignancy**							
**No**	78	12 (15.4)					
			MRI	0.92	[0.86, 0.98]		
			CEUS + MRI	0.93	[0.85, 1.00]	0.01	0.885
**Yes**	19	5 (26.3)					
			MRI	0.73	[0.38, 1.00]		
			CEUS + MRI	0.94	[0.84, 1.00]	0.21	0.138
**Arterial phase hyperenhancement**							
**No**	12	2 (16.7)					
			MRI	0.63	[0, 1.00]		
			CEUS + MRI	0.90	[0.68, 1.00]	0.28	0.323
**Yes**	85	15 (17.7)					
			MRI	0.91	[0.84, 0.98]		
			CEUS + MRI	0.94	[0.86, 1.00]	0.02	0.656
**SI on hepatobiliary phase**							
**Low SI**	65	15 (23.1)					
			MRI	0.84	[0.71, 0.97]		
			CEUS + MRI	0.91	[0.83, 0.99]	0.08	0.251
**Iso to high SI**	32	2 (6.3)					
			MRI	0.98	[0.94, 1.00]		
			CEUS + MRI	0.98	[0.95, 1.00]	0.00	1.000
**Enhancement on Kupffer phase**							
**Hypo-enhancement**	46	16 (34.8)					
			MRI	0.84	[0.72, 0.97]		
			CEUS + MRI	0.87	[0.75, 0.98]	0.03	0.770
**Iso to hyper-enhancement**	51	1 (1.9)					
			MRI	0.98	[0.95, 1.00]		
			CEUS + MRI	0.99	[0.97, 1.00]	0.01	0.270

Note.—*P* value* of difference for AUC was calculated using DeLong’s test for two correlated ROC curves. AUC = area under a ROC curve, SI = signal intensity

In a subgroup analysis based on the presence or absence of underlying extrahepatic malignancy, the presence of arterial-phase hyperenhancement on MRI, the signal intensity on hepatobiliary-phase of MRI, and the degree of enhancement on Kupffer phase, the AUCs increased when CEUS was added to MRI, but the difference was not statistically significant (Table 2).

**Fig 2:**
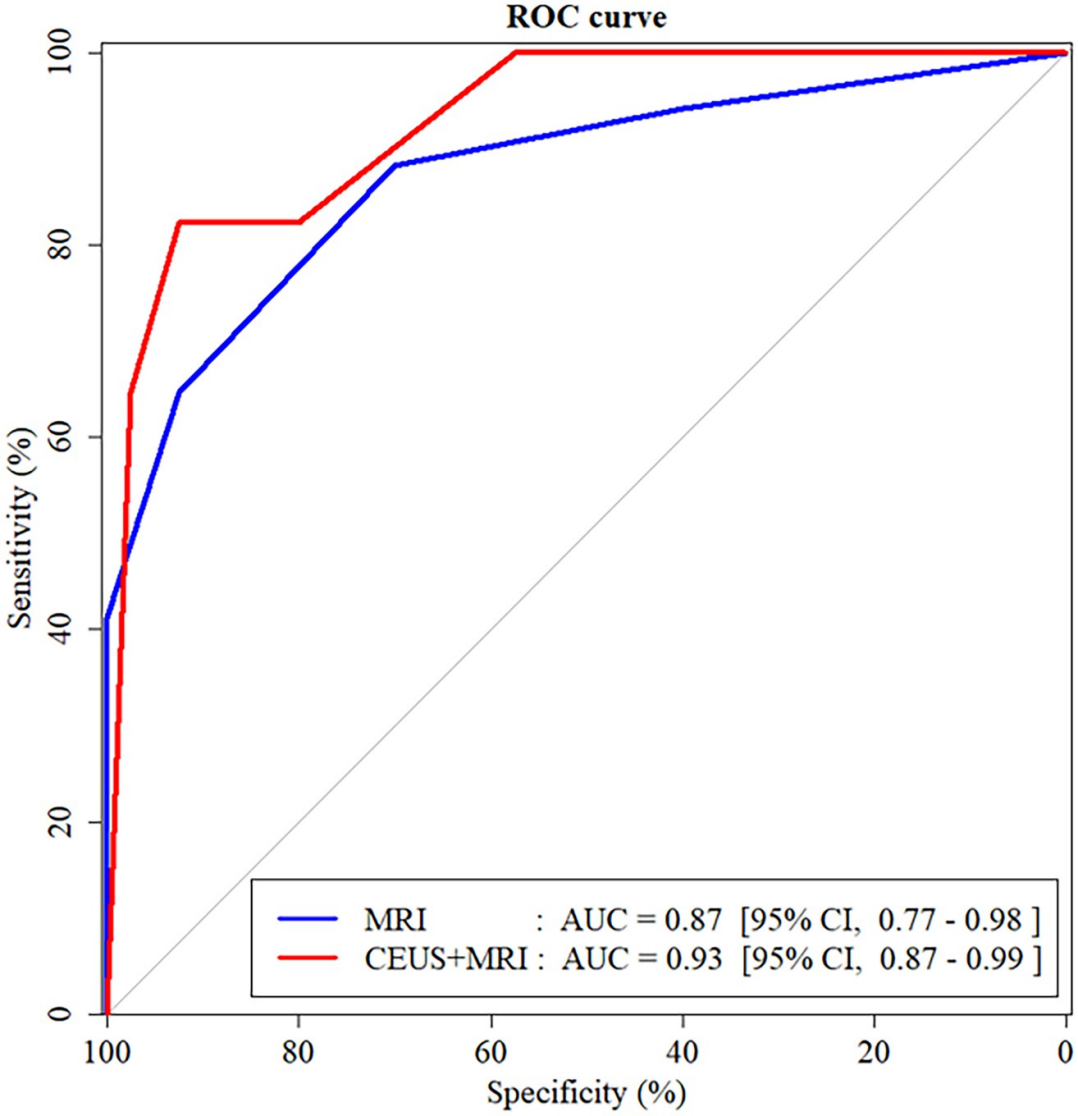
ROC curve analysis for characterizing indeterminate focal liver lesions.

**Fig 3:**
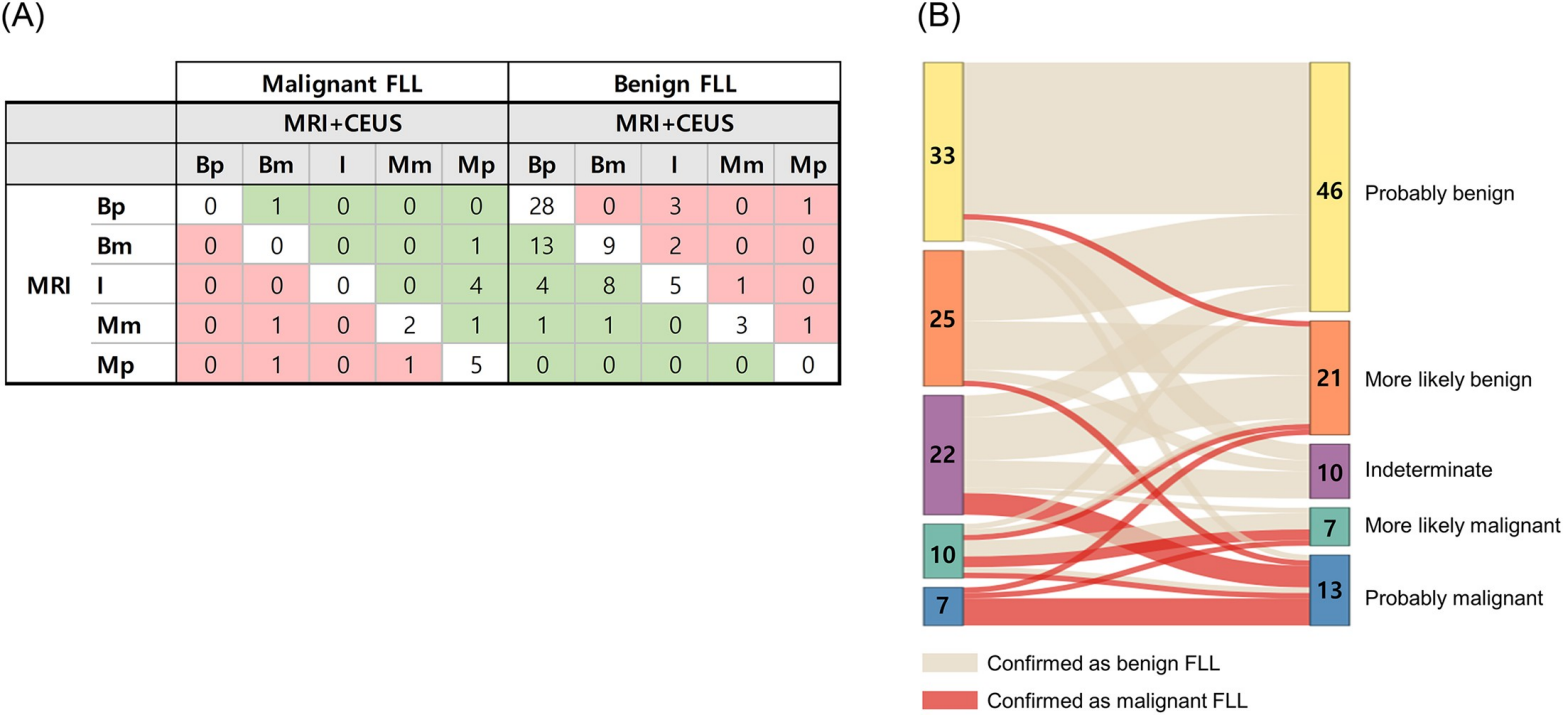
Changes in the FLL categories when adding CEUS to MRI. FLL = focal liver lesion, NRI = net reclassification improvement, Bp = probably benign, Bm = more likely benign, I = indeterminate, Mm = more likely malignant, Mp = probably malignant. **(A)** Reclassification table. In malignant FLLs, the NRI was calculated as (# up—# down) / # total. Therefore, NRI = (7–3) / 17 = 0.235. In benign FLLs, the NRI was calculated as (# down—# up) / # total. Therefore, NRI = (27–8) / 80 = 0.238. For all FLLs, the NRI was calculated as the NRI of malignant FLLs + the NRI of benign FLLs. Therefore, NRI = 0.235 + 0.238 = 0.473. **(B)** The Sankey diagram graphically depicts the changes in category between MRI alone and MRI + CEUS for FLLs. On adding CEUS, the number of FLLs in the indeterminate category decreased (22 FLLs on MRI and 10 FLLs on MRI+CEUS).

**Fig 4:**
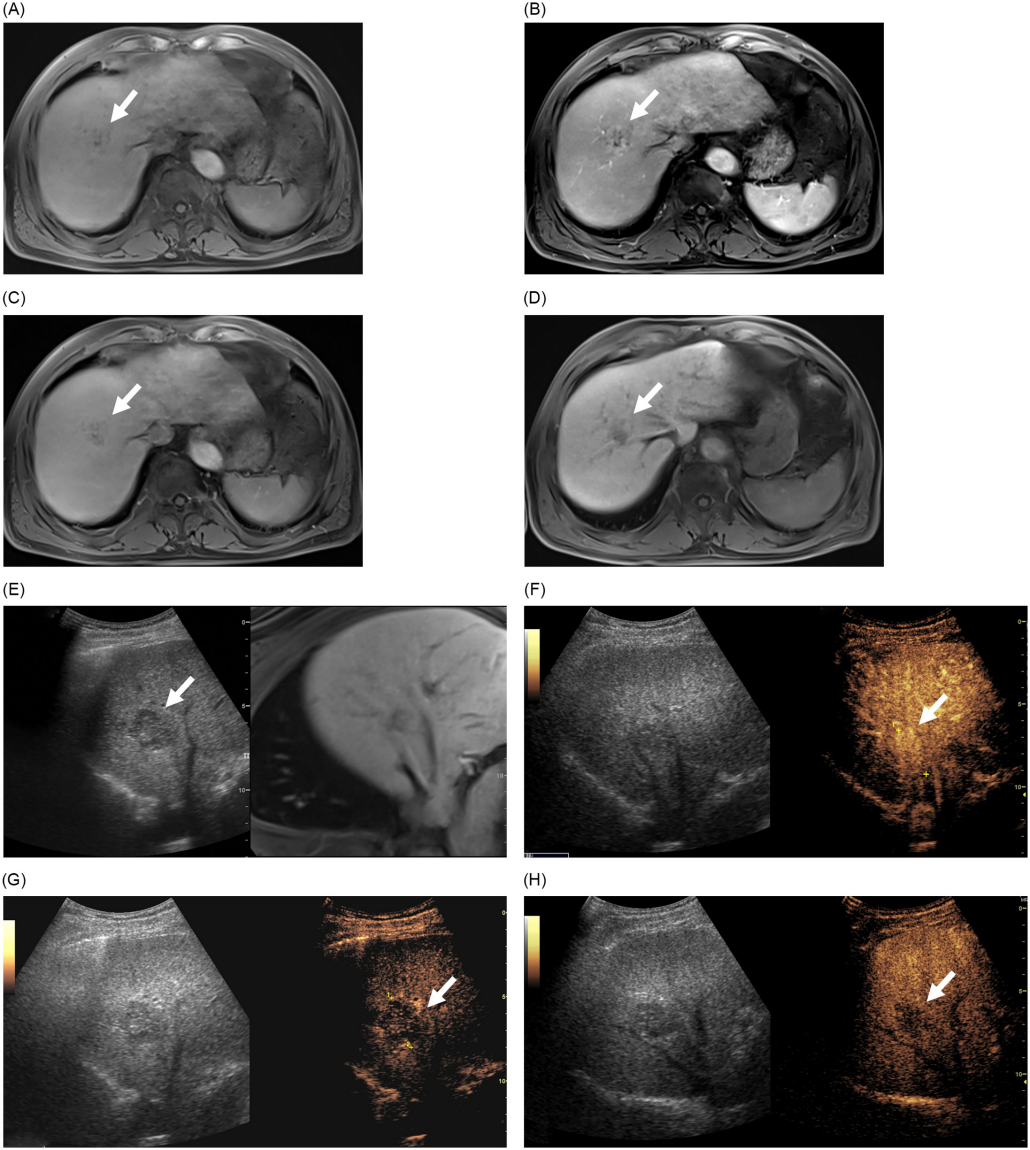
Images in a 72-year-old man with an indeterminate focal liver lesion (arrows) on MRI. An unenhanced MR image shows an irregularly shaped low-to-iso signal intensity lesion in liver segment 8 (A). The lesion shows subtle hyperenhancement on the arterial-phase (B), slightly low signal intensity on the transitional-phase (C), and low-to-iso signal intensity on the hepatobiliary-phase (D). On MRI, the lesion was categorized as indeterminate. Gray scale ultrasound image shows a relatively well-marginated low-echoic mass (E). The lesion shows arterial-phase hyperenhancement (F), late-onset washout (G), and defect on the Kupffer phase (H). On CEUS, it was categorized as probably malignant. The mass was surgically resected and confirmed as a hepatocellular carcinoma.

The changes in FLL categories when CEUS was added to MRI are summarized in Figs 2 and 3. NRI was 0.473 (95% CI, 0.100–0.845; *P* = 0.013). Twenty seven (33.8%, 27/80) benign lesions were appropriately reclassified downwards, and eight (10.0%, 8/80) benign lesions (two hemangiomas, two adenomas, two focal nodular hyperplasia, one possible focal nodular hyperplasia, and one necrotic nodule) were reclassified upwards. Seven (41.2%, 7/17) malignant lesions were appropriately reclassified upwards (Fig 4), and three (17.6%, 3/17) malignant lesions (two HCCs and one metastasis from colon cancer) were reclassified downwards.

When CEUS was added to MRI, one benign lesion (1.3%, 1/80), which was initially categorized as probably benign on MRI, was mis-categorized as probably malignant because the central portion of the lesion appeared hypoechoic on portal venous and late vascular phases of CEUS. Two benign lesions (2.5%, 2/80), which were mis-categorized as more likely malignant on MRI were appropriately re-categorized as more likely benign (no washout on portal venous and late vascular phases of CEUS) and probably benign (showing typical peripheral nodular enhancement and centripetal fill-in enhancement, suggestive of hemangioma). One malignant lesion (5.8%, 1/17), which was mis-categorized as more likely benign on MRI was appropriately re-categorized as probably malignant (showing washout on portal venous and late vascular phases of CEUS), and two malignant lesions (11.8%, 2/17), which were categorized as more likely malignant and probably malignant on MRI were mis-categorized as more likely benign (the two lesions did not show definite washout on portal venous and late vascular phases of CEUS).

## Discussion

The usefulness of CEUS in characterizing indeterminate focal liver lesions on gadoxetic acid-enhanced liver MRI in patients without risk factors for HCC is not well established. This study aims to evaluate the diagnostic value of CEUS with Sonazoid in characterizing indeterminate FLLs on gadoxetic acid-enhanced liver MRI in patients without risk factors for HCC. Our results showed that adding CEUS to MRI did not significantly improve the AUCs for characterizing indeterminate FLLs as benign vs malignant (0.87, 95% CI: 0.77–0.98 for MRI vs 0.93, 95% CI: 0.87–0.99 for CEUS added to MRI, *P* = 0.296).

Previous studies have demonstrated that CEUS is a valuable tool for evaluating FLLs. A meta-analysis has reported that CEUS had an overall sensitivity and specificity of 92% (95%-CI: 91–93%) and 87% (95%-CI: 86–88%), respectively, in diagnosing malignant liver lesions [12]. This meta-analysis included a total of 57 studies, and the types of contrast agents used in each study were as follows: SonoVue (Bracco, Milano, Italy) was used in 39 studies, Levovist (Bayer Schering Pharma, Berlin, Germany) in 12 studies, Sonazoid in 4 studies, Optison (GE Healthcare, Princeton, NJ, USA) in one study, and Definity (Lantheus Medical Imaging, North Billerica, MA, USA) in one study. In the subgroup analysis of this meta-analysis, Sonazoid showed higher diagnostic odds ratio compared to SonoVue (227 vs 119). However, in the prospective study comparing the efficacy of Sonazoid and SonoVue in diagnosing FLLs as benign or malignant, both agents demonstrated similar efficacy [13]. Additionally, CEUS using SonoVue [14–16] or Sonazoid [17, 18] has shown similar to superior performance to that of CT and similar performance to that of MRI in the differential diagnosis of benign and malignant FLLs. Furthermore, some studies have shown that adding CEUS to CT or MRI can improve the diagnostic performance for evaluating FLLs [5, 19]. For instance, a prospective study using SonoVue conducted on 121 biopsy-proven hepatic nodules including 72 HCC lesions demonstrated that the combined assessment of the vascularity of FLLs on CT and CEUS significantly increased the sensitivity for the diagnosis of malignancy (97%) compared with that on CT alone (71–74%) [19]. Another study showed that adding CEUS with Sonazoid can aid in further characterizing Liver Imaging Reporting and Data System category 3 and 4 observations on gadoxetic acid-enhanced liver MRI [5]. However, these studies were performed on patients with risk factors for HCC. The distinctive aspect of our study is that it examines whether CEUS using Sonazoid provides additional benefits over gadoxetic acid-enhanced MRI in patients without risk factors for HCC.

Our study, conducted on patients without risk factors for HCC, yielded results that differed from those of previous studies on the usefulness of CEUS for diagnosing FLLs. Adding CEUS with Sonazoid did not improve the diagnostic performance for characterizing indeterminate FLLs. There are two possible explanations for these findings. First, the presence or absence of risk factors for HCC can affect the differential diagnosis of FLLs. In patients with risk factors for HCC, the evaluation of the lesion focuses on whether the FLL is an HCC. In this case, CEUS may be useful in identifying arterial-phase hyperenhancement or late-onset washout, which are important diagnostic features of HCC. However, in patients without risk factors for HCC, the diseases to be differentiated are more diverse, and the meaning of arterial-phase hyperenhancement or washout can vary. Another possible explanation for our results is that the FLLs included in this study were highly selective. Our liver MRI protocol included various imaging sequences, including diffusion-weighted imaging and hepatobiliary-phase images, which have been shown in previous studies to have a high diagnostic performance for differentiating between benign and malignant FLLs [20–22]. Thus, the FLLs included in our study, which could not be differentiated even by using MRI with such high a diagnostic performance, might have rare or atypical imaging findings.

In our study, 33.8% (27/80) of the benign lesions and 41.2% (7/17) of the malignant lesions were correctly reclassified after adding CEUS. In contrast, 10.0% (8/80) of the benign lesions and 17.6% (3/17) of the malignant lesions were incorrectly reclassified. These results indicated that although CEUS did not improve the overall diagnostic performance measured by AUC, it did enhance the radiologist’s confidence in accurately classifying benign lesions as benign and malignant lesions as malignant.

Subgroup analysis based on underlying extrahepatic malignancy, the presence of arterial-phase hyperenhancement on MRI, and hepatobiliary-phase signal intensity did not show a statistically significant improvement in the diagnostic performance by adding CEUS. However, in the subgroup without arterial-phase hyperenhancement on MRI, the AUC showed the greatest difference (0.63 for MRI vs 0.90 for CEUS added to MRI, *P* = 0.324). In this subgroup, identification of arterial-phase hyperenhancement for malignant lesions and reconfirming the absence of arterial-phase hyperenhancement or identification of peripheral nodular enhancement for benign lesions on CEUS helped improve diagnostic performance. Although this difference was not statistically significant, there is a possibility that CEUS may be helpful for this subgroup. However, further research in this issue is required.

Our study had several limitations. First, it was a single-center study, which might have limited the generalizability of our findings to other institutions with different patient populations and disease entities. Second, it is retrospective study, and FLLs were categorized retrospectively. Although the final diagnosis of FLLs was blinded at the time of image review, and there was a time interval between the CEUS examination and the image review for FLLs categorization, the reviewers might have remembered some FLLs, which might have influenced the outcome. Third, although we screened all the patients who underwent CEUS at our institution, the sample size was small, which might have limited our study from detecting significant differences. Therefore, further research with larger sample sizes and prospective study designs is required to confirm our findings.

In conclusion, performing CEUS using Sonazoid does not significantly improve the overall diagnostic performance for characterizing indeterminate FLLs on gadoxetic acid-enhanced liver MRI in patients without risk factors for HCC. However, CEUS may increase radiologist’s confidence in classifying FLLs. Further research is required to better understand the potential benefits of CEUS in specific subgroups of patients with indeterminate FLLs.

## Supporting information

S1 Data The original data. (XLSX)

## Abbreviations

AUC: area under the ROC curve
CEUS: contrast-enhanced ultrasonography
CI: confidence interval
FLL: focal liver lesion
HCC: hepatocellular carcinoma
NRI: net reclassification improvement
ROC: receiver operating characteristic
USCA: Ultrasound contrast agent
